# Characterisation of the Convective Hot-Air Drying and Vacuum Microwave Drying of *Cassia alata*: Antioxidant Activity, Essential Oil Volatile Composition and Quality Studies

**DOI:** 10.3390/molecules24081625

**Published:** 2019-04-24

**Authors:** Lisa Yen Wen Chua, Bee Lin Chua, Adam Figiel, Chien Hwa Chong, Aneta Wojdyło, Antoni Szumny, Krzysztof Lech

**Affiliations:** 1 School of Engineering, Taylor’s University, Lakeside Campus, No. 1, Jalan Taylor’s, Subang Jaya, Selangor 47500, Malaysia; lisacyw92@gmail.com; 2 Institute of Agricultural Engineering, Wrocław University of Environmental and Life Sciences, 37a Chełmońskiego Street, 51-630 Wrocław, Poland; adam.figiel@upwr.edu.pl (A.F.); krzysztof.lech@upwr.edu.pl (K.L.); 3 School of Engineering and Physical Sciences, Heriot-Watt University Malaysia, No. 1 Jalan Venna P5/2 Precinct 5, Putrajaya 62200, Malaysia; chien_hwa.chong@hw.ac.uk; 4 Department of Fruit, Vegetable and Plant Nutraceutical Technology, Wrocław University of Environmental and Life Sciences, 37 Chełmońskiego Street, 51-630 Wrocław, Poland; aneta.wojdylo@upwr.edu.pl; 5 Department of Chemistry, Wrocław University of Environmental and Life Sciences, Norwida 25, 53-375 Wrocław, Poland; antoni.szumny@upwr.edu.pl

**Keywords:** *Cassia alata*, vacuum microwave, antioxidant activity, essential oil volatile composition, phytosterol, drying technology

## Abstract

The preservation of active constituents in *Cassia alata* through the removal of moisture is crucial in producing a final product with high antioxidant activity. This study aims to determine the influences of various drying methods and drying conditions on the antioxidant activity, volatiles and phytosterols content of *C. alata*. The drying methods used were convective drying (CD) at 40 °C, 50 °C and 60 °C; freeze drying; vacuum microwave drying (VMD) at 6, 9 and 12 W/g; and two-stage convective pre-drying followed by vacuum microwave finish drying (CPD-VMFD) at 50 °C and 9 W/g. The drying kinetics of *C. alata* are best described by the thin-layer model (modified Page model). The highest antioxidant activity, TPC and volatile concentration were achieved with CD at 40 °C. GC–MS analysis identified the presence of 51 volatiles, which were mostly present in all samples but with quantitative variation. The dominant volatiles in fresh *C. alata* are 2-hexenal (60.28 mg 100 g^−1^ db), 1-hexanol (18.70 mg 100 g^−1^ db) and salicylic acid (15.05 mg 100 g^−1^ db). The concentration of phytosterols in fresh sample was 3647.48 mg 100 g^−1^ db, and the major phytosterols present in fresh and dried samples were β-sitosterol (1162.24 mg 100 g^−1^ db). CPD-VMFD was effective in ensuring the preservation of higher phytosterol content in comparison with CD at 50 °C. The final recommendation of a suitable drying method to dehydrate *C. alata* leaves is CD at 40 °C.

## 1. Introduction

The majority of the world population has used medicinal plants in some ways as their first source of treatment as remedy for infectious and non-infectious diseases. Many of these medicinal plants have been researched and have garnered scientific evidence to be considered and used in general medical practice. A large number of diseases are caused by oxidative stress that can elicit cellular and tissue damage—a result of increased levels of free radicals or reactive oxygen species (ROS). Deficiency of antioxidants or overproduction of ROS in the body can cause the disturbance in the equilibrium between ROS formation and elimination, favouring the upsurge of ROS, which results in oxidative stress [1]. Hence, discovering plants that exhibit antioxidant effect that can be a potential source of antioxidants is necessary. 

*Cassia alata* (Leguminosae) is a shrub native to Southeast Asia, Northern Australia, Latin America, Fiji and Africa and can be found widespread in tropical regions, cultivated for medicinal purposes [2,3]. *C. alata* is known as gelenggang in Malaysia, ringworm bush in Australia, mbai ni thangi in Fiji and te’elango in Tango [2]. This shrub can grow up to 3 m in height. The leaves are 30–40 cm long, pinnately compound, have 6–12 pairs of broad leaflets, blunt at the tip portion, with unequal base and are 15 cm long and 8 cm wide. The flowers are golden yellow, roundish in axillary racemes that are 3–4 cm wide and 20–30 cm long [4]. *C. alata* is widely used in folk medicine and home remedies for treating common ailments, such as ring worm, skin diseases, rheumatism, diabetes, stomach pain, constipation and gonorrhoea [3,5]. Numerous past reports on the promising antioxidant effect shown by *C. alata* extract have prompted the investigation of the influence of drying methods on the antioxidant activity and chemical constituents [6,7,8]. The leaf extract of *C. alata* is reported to contain phenols, flavonoids and anthraquinones, which is believed to contribute to the strong antioxidant effect of the plant extract [6].

Freshly harvested medicinal plants have high water content and are thus categorised as perishable commodities. Unfavourable enzymatic and metabolic activities are likely to occur due to high water content, in addition to the increased susceptibility to microbial growth [9]. Bioactive content may reduce considerably in the raw material between the period of harvesting and processing. Effective preservation via the removal of moisture is needed to inactivate enzymatic degradation and chemical and metabolic reactions [10]. Drying operations have been used in the food processing industry for the preservation of final product. The primary aim of drying is to reduce moisture in food to a level that can avoid microbial spoilage. However, drying can also be seen as a process that retains important constituents in raw herbs with nutraceutical properties [11]. 

Convective drying (CD) remains the most widely used drying method for reducing moisture from fruits, vegetables and medicinal plants. However, CD methods have several limitations; for example, they are a low energy efficiency drying method and may not necessarily produce dried medicinal herbs with high retention of bioactive compounds [9]. In addition, CD is a surface heating method. Firstly, water evaporates from the surface of food material, and the subsequent moisture removal from the inner regions of raw material is driven by a moisture concentration gradient. Diffusion of moisture from the centre areas of the material to the surface is slow and rate limiting, thereby resulting in low drying rate at the final stage of drying [12]. Prolonged drying durations can result in extensive quality deterioration in the final dried product [13]. At the same time, bioactive constituent loss may also be contributed by the long exposure of plants to oxygen during the CD process [14].

The introduction and application of hybrid drying technologies are necessary to overcome the limitations of conventional drying methods whilst considering the sensitivity of bioactive constituents in medicinal herbs to thermal degradation and oxidation. Combined drying methods are perceived as more versatile because the advantages of two or more drying methods may offer better drying outcomes than an individual approach [9]. Vacuum microwave drying (VMD) is a combined microwave heating and vacuum drying method. This combination is deemed appropriate for drying process to occur in a reduced pressure environment, thereby lowering oxidation reactions and lowering the boiling point of water. During the process of VMD, microwaves will penetrate the plant material and cause water to be heated up via dipole rotation and ionic conduction, thereby causing water vaporisation at a low temperature under a reduced pressure condition [11]. In addition to increasing the drying rate with VMD’s volumetric heating, the lower pressures used can induce rapid water evaporation from plant materials at a low drying temperature, thereby minimising possible chemical alterations in plant material by high temperatures [15].

The problem with VMD is that high water evaporation from the plant material at the initial stage of drying may exceed the vacuum pump’s capacity; therefore, the material load has to be reduced, or a large vacuum pump installation is often required [11,16]. Installing a large-scale vacuum pump requires a large capital and can potentially incur a large processing cost. Convective pre-drying followed by vacuum microwave finish drying (CPD-VMFD) is a two-stage combined drying method in which the raw material is pre-dried using CD and finish dried with VMD. This method has been used in numerous studies being a cheaper alternative of VMD in terms of capital expenditures and operating costs. The high retention of bioactive constituents in various biomaterials has also been reported with this two-stage method [17].

The present study aims to evaluate the influences of various drying methods, conventional drying method, CD, new drying technologies (namely, VMD and CPD-VMFD) and the respective drying intensities on the total phenolic content, antioxidant activity, volatiles and phytosterol content. The fatty acid profile of the plants was ascertained to understand the active constituents contained in *C. alata*. The drying kinetics was modelled with three common thin-layer drying models to integrate the experimentally acquired data into industrial applications, thereby providing the basis for the understanding of the unique drying characteristics of *C. alata*. Drying is considered the most energy-intensive operation in the food industry. Thus, this study also determined the relationship of the drying kinetics of *C. alata* with the corresponding energy input of a drying operation. The specific energy consumptions were used to provide an estimate of the cost-effectiveness of the drying of *C. alata*, which has yet to be reported. Colour properties and water activities of fresh and dried samples, which serve as quality indicators, were also determined.

## 2. Results and Discussion

### 2.1. Drying Kinetics

**Figure 1:**
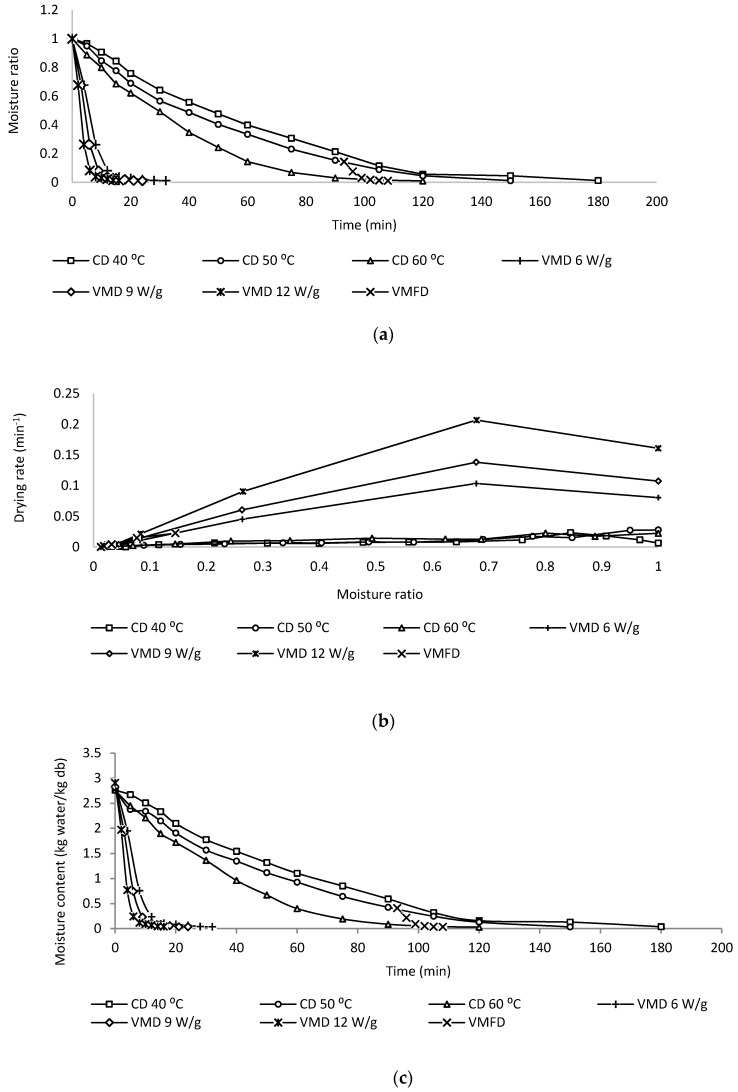
(**a**) Relationship between moisture ration (MR) and time. (**b**) Drying rate against MR. (**c**) Relationship between moisture content and time for the drying of *C. alata* at convective drying (CDs) at 40 °C, 50 °C and 60 °C, vacuum microwave drying (VMD) at 6, 9 and 12 W/g and convective pre-drying followed by vacuum microwave finish drying (CPD-VMFD) at 50 °C and 9 W/g.

Figure 1a shows the change of moisture ratio (MR) with time, Figure 1b shows the relationship between the drying rate and MR and Figure 1c shows the change of moisture content with time of *C. alata* dried using CD at 40 °C, 50 °C and 60 °C, VMD at 6 W/g, 9 W/g and 12 W/g and CPD-VMFD at 50 °C and 9 W/g. As shown in Figure 1a,c, the drying duration of CD is the longest as convective method uses surface heating, and surface moisture is removed efficiently; however, the slow diffusion of moisture from the internal region is rate limiting. A rapid moisture removal in the initial stage of CD is typical, and moisture removal will progressively reduce with time. With VMD, the volumetric heating of microwaves addresses the limitation of CD because the water removal from the centre region is fast, thereby resulting in higher drying rates than CD methods. A higher drying rate during the initial period of VMD is often observed as a larger microwave radiation is absorbed due to a higher moisture level in leaves. As drying progresses, lower moisture content in leaves corresponds to the lower microwave absorption, thereby lowering the drying rate. In the combined method CPD-VMFD, CPD is effective in removing moisture during the initial drying process whilst VMFD assists in the removal of residual moisture, which is strongly bound to the plant cellular structure, thereby shortening CD from 150 min to 105 min.

With regard to CD and VMD, high drying intensities resulted in reduced drying durations and high drying rates. Increasing hot-air temperature for CD from 40 °C to 60 °C reduced drying time from 180 min to 120 min. Meanwhile, for VMD, increasing microwave wattage from 6 W/g to 12 W/g reduced drying time from 32 min to 16 min. The trend of MR with time of CD and VMD at different drying intensities is similar (Figure 1a). Drying kinetics of *C. alata* dried using CD, VMD and CPD-VMFD are best described by an exponential function (modified Page model).

(1) $$MR=a\cdot \exp\;\left(-k\cdot t^{n}\right).\;$$

### 2.2. Water Activity Analysis

Water activity *a_w_* measures the degree of water that is bound to a food product and predicts the product’s safety and stability with regard to microbial growth and the rates of chemical and biochemical reaction rates. The *a_w_* of fresh and dried *C. alata* leaves and the final moisture content of *C. alata* samples dried with CD, VMD and CPD–VMFD are shown in Table 1. The *a_w_* of the dried samples ranged from 0.1291 to 0.2836. The CD at 60 °C samples had the lowest final moisture content and significantly lower *a_w_* than other dried samples, which could be attributed to the high hot-air temperature applied and the longer drying duration than VMD and CPD-VMFD, thereby ensuring low residual moisture in the sample. Increasing temperature in CD resulted in lower *a_w_* and final moisture content. However, the application of different levels of microwave wattage showed no evident trends. Preferably, dried products should attain a low *a_w_* ranging from 0.60 to 0.80 because food spoilage bacteria and most moulds are inhibited below the *a_w_* of 0.91 and 0.80, respectively [18]. In the present study, all of the drying treatments ensured microbiologically safe products. The determination of *a_w_* is also important in the prediction of biochemical reactions that may occur, knowing that Maillard reaction takes place at an optimum a_w_ of 0.65 [19].

### 2.3. Specific Energy Consumption

**Figure 2:**
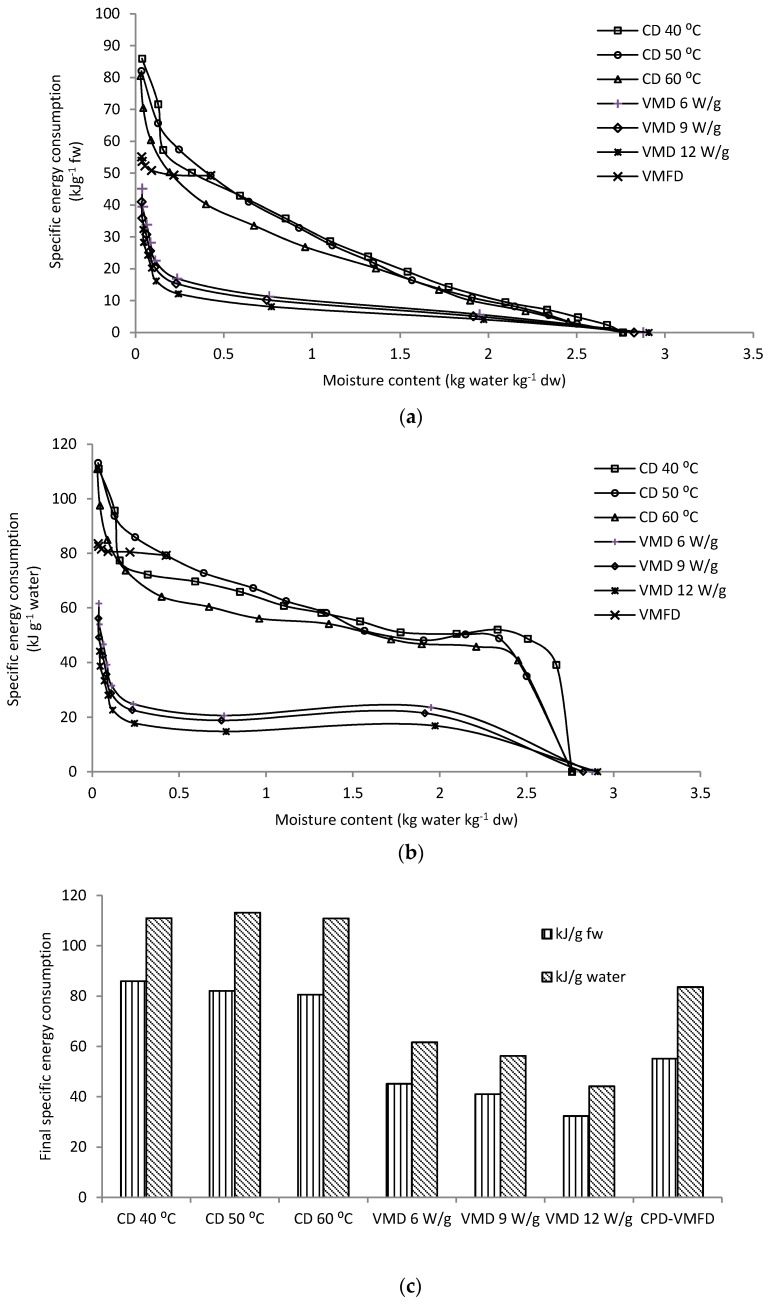
(**a**) Specific energy consumption of *C. alata* leaves per gram of fresh weight dried using CD, VMD and CPD-VMFD. (**b**) Specific energy consumption of *C. alata* leaves per gram of water removed using CD, VMD and CPD-VMFD. (**c**) Final specific energy consumptions of CD, VMD and CPD-VMFD of *C. alata* leaves.

The profiles of specific energy consumption shown in Figure 2a,b represent the amount of energy required to remove moisture from fresh leaves per 1 g of fresh weight and 1 g of water, respectively. Evidently, the shape of the curves suggests that drastic increments of specific energy consumptions are noted at the last stages of the drying process, irrespective of drying methods used. This result indicates that a considerable amount of energy is required to remove the same amount of water compared with the initial drying stages at a specific drying parameter. This behaviour is common in plant products with osmotic cellular structure that functions to limit the loss of moisture from plant tissues [18].

Figure 2c presents the final specific energy consumptions of CD, VMD and CPD-VMFD. VMD achieved the lowest energy consumptions compared with CD methods, particularly at a high microwave wattage. However, practicality wise, the installation of a large-scale microwave vacuum dryer is costly. Combined drying, involving CD followed by VMFD, is highly recommended for industrial processes considering cost-effectiveness. Therefore, CPD-VMFD is deemed as a compromise between CD and VMD methods in terms of energy consumption requirement. In this study, CPD-VMFD achieved lower final specific energy consumption (55.13 kJ/g fresh weight and 83.60 kJ/g water) than CD at 50 °C (82.06 kJ/g fresh weight and 113.15 kJ/g water) and slightly higher final specific energy consumption than VMD. CPD-VMFD achieved reduction in the final energy consumptions by 32.82% (kJ/g fresh weight) and 26.12% (kJ/g water) in comparison with CD at 50 °C.

### 2.4. Colour Analysis

Dried leaves often appear slightly olive-brownish because drying results in the disruption of the cytoplasmic membrane, which leads to the release of cellular acids that degrade chlorophylls to pheophytins, with olive-brownish and greenish-coloured derivatives [20]. Table 2 shows the colour parameters of fresh and dried samples of *C. alata* affected by different drying methods and conditions. In this study, freeze-dried samples had the lightest colour, indicated by a high L* value, with the greenest (lowest a* value) and yellowest colour (highest b* value) amongst the other dried samples. Generally, convective-dried samples were darker in colour compared with VMD and CPD-VMFD samples. The microwave treatment proved effective in retaining the green colour and lightness of herbs due to the accelerated drying process coupled with a reduced oxygen condition. However, a low hot-air temperature of 40 °C was also effective in preserving the green colour of the leaves. A high temperature applied during CD was observed to increase the degradation of chlorophylls (shown by the higher a* values), although the drying duration was reduced in comparison with CD at 40 °C. FD achieved the highest b* value (yellow). The low-temperature drying process of FD is effective in preserving carotenoids, which are heat-sensitive. Moreover, VMD and CPD-VMFD were also effective in retaining carotenoids than CD. Fresh samples appear darker with less yellow colour intensity because the high moisture in leaves may have led to the destruction of plant pigments during the time frame between the harvest process and analysis.

### 2.5. Antioxidant Activity and Total Phenolic Content (TPC) Analysis

The analysis of antioxidant activity and TPC suggests a considerable reduction in radicals of 2,2′-azinobis(3-ethylbenzthiazoline-6-sulfonic acid) (ABTS), and Fe^+3^ to Fe^+2^ by FRAP methods and TPC values were observed after drying (Table 3). Amongst the drying treatments, the highest antioxidant activity and TPC values were observed for CD at 40 °C and freeze-dried samples. *C. alata* dehydrated using CD at 40 °C achieved higher antioxidant activity in comparison with VMD methods, although the ABTS and FRAP values of CD at 40 °C was not significantly different than VMD methods (Table 3). This observation could be the result of polyphenols being exposed to oxygenated condition. Polyphenols in an intermediate oxidised state have been reported to show greater antioxidant effect compared with their non-oxidised state [21]. In addition, high-temperature stabilisation process could result in the production of Maillard reaction products (MRPs) formed from Maillard reaction in convective-dried samples. MRPs are known to exhibit antioxidant effect, often by chain breaking mechanism [22]. The assumption that MRPs could contribute to the overall increase in antioxidant activity of CD at 40 °C samples is supported by the lower L* values of convective-dried samples compared with VMD samples, which indicates more browning (Table 2). However, only at a low hot-air temperature (40 °C) was it effective in preserving the antioxidant properties of *C. alata* because the antioxidant activities were seen to decline at 50 °C and 60 °C. Samples dried using CD at 60 °C produced the lowest ABTS, FRAP and TPC values. The high extent of loss in antioxidant values and phenolic content could be attributed to the high degradative temperature at 60 °C. As for VMD, moderate microwave wattage of 9 W/g was advantageous in achieving high antioxidant effect and TPC. Meanwhile, CPD-VMFD produced intermediate antioxidant activity and TPC values.

The TPC values are shown in Table 3. All drying treatments resulted in large reduction in TPC values, with CD at 60 °C obtaining the lowest TPC value, whilst CD at 40 °C achieved the highest TPC. Low TPC values of the heat-treated samples may be caused by the oxidative and thermal degradation of phenolic compounds. The results showed that CD at 40 °C could preserve phenolic compounds better than freeze drying (FD), contrary to the understanding that FD, a low-temperature dehydration method, could preserve high amounts of phenolic compounds. However, a previous report has suggested that FD treatment may not have deactivated degradative enzymes completely because of the low-temperature dehydration process. This could lead to the reactivation of degradative enzymes when freeze-dried samples are exposed to moisture in the air [23]. This is supported by the result obtained by Li et al. [24], which investigated the thermal inactivation kinetics of polyphenol oxidase (PPO) of chicory leaves. It was found that the residual activities of PPO at the end of hot air drying was considerably lower than that of FD, which indicates that hot air drying was more effective in the inactivation of PPO compared to FD, inhibiting enzymatic oxidative reactions to an extent. It was suggested that enzymatic oxidative reaction is more likely to occur during FD as the formation of ice crystals in the tissue matrix of the raw material will lead to cell rupture, subsequently exposing phenolic species to oxidative conditions. However, most studies have shown that FD is able to preserve phenolic compounds better than hot air drying, in the case of oregano leaves [25], strawberry [21] and citrus fruits [26]. Therefore, the influence of a particular drying method on the preservation of phenolic compounds is difficult to predict and is dependent on the specific compounds and the particular plant material involved.

**Figure 3:**
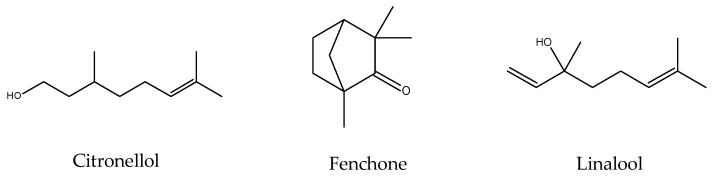
Chemical structures of citronellol, fenchone and linalool.

Overall, the antioxidant activity values failed to correlate well with TPC values. Thus, antioxidants other than phenolic compounds could have contributed to the total antioxidant activity (terpene compounds) contained in volatiles, phytosterols and fatty acid constituents in *C. alata*. For example, terpenes contained in *C. alata* (such as citronellol, linalool and fenchone; Figure 3) are amongst the identified terpenes that could contribute to the total antioxidant activity. This result is based on the fact that terpenes have been reported to be potential antioxidant compounds because of their capability to interact with free radicals. The antioxidant potential of terpenes is suggested to be similar to flavonoids, and their antioxidant effect depends on the structural characteristics such as the presence of hydroxyl groups, multiple bonds, conjugated systems and phenolic structure [27].

The interactions amongst antioxidants resulting in higher antioxidant effect has been widely reported and acknowledged because enhanced antioxidant effect is evident when antioxidants are combined than when isolated [28,29,30]. This phenomenon is termed as synergism, which results in the higher net interactive antioxidant activity compared with the sum of antioxidant activities exerted by individual antioxidants. Synergism occurs when (i) an antioxidant can donate electrons to other antioxidants, thereby facilitating their regeneration; (ii) when an antioxidant exerts protection on another antioxidant by sacrificial self-oxidation; and when (iii) two or more antioxidants exhibit antioxidant mechanisms [31]. However, future studies are needed to correctly ascertain the interactions between antioxidants in *C. alata* and it remains to be confirmed if certain combinations of primary antioxidants present in *C. alata* will lead to an overall greater antioxidant effect. The type of interactions between antioxidants is important to determine if the extract of *C. alata* is to be used as a potential source of antioxidant compounds. Future studies should also include development of drying procedures by incorporation of other methods of drying or some pre-treatment in order to preserve the largest possible amount of native compounds responsible for bioactive potential.

### 2.6. Analysis of Volatile Content

Volatile constituents of fresh and dried *C. alata*, which were extracted using HS-SPME and analysed with GC–MS, revealed the presence of 50 volatile compounds. Table 4 presents the identified volatile compounds and the respective concentrations. The total concentration of volatiles in fresh *C. alata* was 254.79 mg 100 g^−1^ db, and the major volatiles were 2-hexenal (60.28 mg 100 g^−1^ db), 1-hexanol (18.70 mg 100 g^−1^ db) and salicylic acid (15.05 mg 100 g^−1^ db). The analysis of volatile compounds showed that high losses of volatile content followed after drying; however, some drying methods could retain higher volatile content.

Majority of volatile compounds were present in the dried sample but differed in their concentrations, with respect to the different drying methods and conditions. In the case of FD, losses of volatiles were higher than expected; however, the loss of volatile constituents during FD was linked to the sublimation of water during the process. The pressure in the chamber should be increased slightly to reduce the loss of volatiles during FD, as reported by Calin-Sanchez et al. (2011) who stated that losses of volatiles is a result of the reduction in the pressure of drying chamber [32]. 

With regard to CD, huge differences in volatile content were observed when hot-air temperature was varied. A low application of temperature (CD at 40 °C) led to a high retention of volatiles. The total concentration of volatiles decreased from 61.85 mg 100 g^−1^ db to 34.55 mg 100 g^−1^ db when temperature was increased from 40 °C to 60 °C. This trend is noted considering individual volatile compounds—with concentrations being high when low temperature was applied. However, the concentration of some volatiles, such as β-cubebene, 2-undecanone and citronellol, failed to follow this trend. The respective concentrations of these volatiles at 60 °C were higher in comparison with 50 °C. With VMD, increasing the microwave wattage from 6 W/g to 12 W/g in VMD resulted in the increase of volatile content from 11.91 mg 100 g^−1^ db to 55.67 mg 100 g^−1^ db. A high microwave wattage was beneficial because the increased drying intensity effectively reduced the total drying time, and volatiles were less exposed to the high degradative temperature.

### 2.7. Phytosterol Analysis

Preliminary studies have shown that CPD-VMFD and CD 50 °C guaranteed high phytosterol yield. Thus, CPD-VMFD and CD at 50 °C were used to determine the effect of drying on the concentration of phytosterols in the present study. Table 5 shows the phytosterols in *C. alata* and their respective concentrations. The total concentrations of phytosterols in fresh sample amounted to 3647.48 mg 100 g^−1^ db. The major phytosterols present in fresh and dried samples were β-sitosterol, followed by stigmasterol and campesterol. CPD-VMFD was expected to retain a higher concentration of phytosterols (809.56 mg 100 g^−1^ db) compared with CD at 50 °C (757.07 mg 100 g^−1^ db) because the low temperature and reduced oxygen condition during the microwave treatment during VMFD could have lessen the thermo-oxidation of phytosterols. This result coincides with the findings of Soupas et al. (2004) and Rudzińska, Przybylski and Wa̧sowicz (2009), who reported that high drying temperature and prolonged drying time can decrease phytosterol concentration [33,34]. Numerous factors, such as process conditions (including heating time, temperature and phytosterol chemical structure), could affect the oxidative stability of the compound. However, the high losses of phytosterols in *C. alata* in comparison with the phytosterol content of fresh sample were probable due to the oxidation process and heat degradation.

### 2.8. Fatty Acid Analysis

Thermal drying has been reported to have a minor influence on the concentration of fatty acids because the structure of fatty acids remained stable at a high temperature of 325 °C [35]. In another study, negligible amount of fatty acids was degraded when thermally treated at 90 °C for 30 min [36]. Therefore, in the present study, the drying temperatures applied could be assumed to possess minor degradative effect. Table 6 shows the identified fatty acids and their relative abundance determined with GC–MS analysis. α-Linolenic acid was the major fatty acid present, followed by linoleic and palmitic acids. α-Linolenic acid is an antioxidant, a plant-derived omega-3 fatty acid and is known for its role in mediating cardiovascular disease [37].

## 3. Materials and Methods

### 3.1. Chemicals and Reagents

ABTS, FRAP reagent, acetate buffer, Folin–Ciocalteu reagent, gallic acid, sodium carbonate, acetic acid, 2,4,6-tripyridyl-1,3,5-triazine (TPTZ), potassium persulfate, 6-hydroxy-2,5,7,8-tetramethylchroman-2-carboxylic acid (Trolox), BF_3_/MeOH, potassium hydroxide, *p*-methoxyphenol, chloroform/(folsch), hexane, sodium chloride, methanol, magnesium sulphate, pyridine, hydrochloric acid (HCl) and *N*,*O*-bis(trimethylsilyl)trifluoroacetamide (BSTFA) were purchased from Sigma-Aldrich (Steinheim, Germany).

### 3.2. Plant Material Preparation

The leaves of *C. alata* were purchased from TKC herbal plantation (Seremban, Malaysia). Identification was confirmed at the Forest Research Institute of Malaysia with voucher specimen: 047/17. The initial moisture content in the range from 2.761 kg water/kg dw to 3.645 kg water/kg dw was determined by drying fresh leaves to a constant mass using a vacuum oven dryer at a temperature of 60 °C for 24 h. In this study, *C. alata* leaves were segregated into 40 g portions and dried using four drying methods. Dying process was stopped when the mass loss of consecutive dried leaves was 0.05 g or less. A blender was used to ground the dried leaves into a fine powder.

### 3.3. Drying Methods

#### 3.3.1. CD

Leaves of *C. alata* were dried using a convective hot-air dryer designed and constructed at the Institute of Agricultural Engineering (Wroclaw, Poland) at 40 °C, 50 °C and 60 °C with an air velocity of 0.5 ms^−1^.

#### 3.3.2. VMD

The leaves were dried with a vacuum microwave dryer (Plazmatronika, Wroclaw, Poland) equipped with a cylindrical drum, connected to a vacuum system and was rotated at 6 rpm throughout the course of drying. The dryer was equipped with a fan that provided a stream of air with a velocity of 1 ms^−1^ to avoid local overheating. Three microwave power levels (6, 9 and 12 W/g) and pressures ranging from 4 kPa to 6 kPa was applied in this study. Leaves were periodically taken out from the dryer to measure the maximum temperature of dried leaves with an i50 infrared camera (Flir Systems AB, Portland, OR, USA).

#### 3.3.3. Two-Stage Drying: CPD-VMFD

Leaves were pre-dried with a convective hot-air dryer similar to Section 3.3.1 at 50 °C and drying was stopped after 90 min at a moisture content of 0.425 kg water kg^−1^ dw. The leaves were finished and dried with a vacuum microwave dryer (Plazmatronika, Wroclaw, Poland) at 9 W/g.

#### 3.3.4. FD

Leaves were freeze-dried using a freeze dryer OE-950 (Labor, MIM, Budapest, Hungary) at a temperature of −60 °C and pressure of 65 Pa. The heating plate reached a temperature of 30 °C for sublimation process to occur. Freeze-dried samples were used as control samples in this study.

### 3.4. Modelling of Drying Kinetics

The drying kinetics of *C. alata* leaves dried with CD, VMD and CPD-VMFD were based on the mass losses of samples. The MR of *C. alata* leaves was determined as follows:

(2) $$MR=\;\frac{M\left(t\right)-\;M_{e}}{M_{0}-M_{e}},$$

where M(t)  is the moisture content (kg water/kg dry matter (dm)) after drying time *t*, $M_{e}$ is the equilibrium moisture content (kg water/kg dm) and $M_{0}$ is the initial moisture content (kg water/kg dm). The equilibrium moisture content $M_{e}$ was determined at the final stage of drying as an asymptotic value of the function fitted to the experimental points by using Table Curve 2D Windows v2.03 (Jandel Scientific Software, San Rafael, CA, USA). The drying curves were fitted to three commonly mentioned thin-layer drying models (Table 7). The best-fitted model that best described the drying processes was determined on the basis of the highest value of coefficient of determination *R^2^* and lowest root mean square error (RMSE).

### 3.5. Water Activity Analysis

The water activity of *C. alata* leaves was determined using a water activity meter (Aqualab 4TE, Pullman, WA, USA) and analysed at an average temperature of 24.9 ± 0.05 °C.

### 3.6. Determination of Energy Consumption

#### 3.6.1. Energy Consumption 

The energy consumptions during the processes of CD ($E_{C}$) and VMD ($E_{VM}$) were calculated on the basis of Equations (3) and (4) [18].

(3) $$E_{C}=\left(\frac{N_{f}}{6}+N_{h}\right)\times t,$$

where $N_{f}$ (kW) is the power consumption of the fan supplying air to the six pipes that were fixed with electric heaters requiring a power consumption of $N_{h}$·(kW), and t is the drying duration (s).

(4) $$E_{VM}=\left(\frac{N_{M}}{\eta_{M}}+N_{V}+N_{e}\right)\times t,$$

where $N_{M}$ is the output power of the magnetrons (kW), $\eta_{M}$ is the efficiency of the magnetrons, $N_{V}$ is the power consumption (kW) of the vacuum pump, $N_{e}$ is the power consumption of the engine (kW) that functions to rotate the container in the dryer and t is the drying duration (s).

#### 3.6.2. Specific Energy Consumption

The specific energies consumed by the process of CD, VMD and CPD-VMFD were determined on the basis of the ratio of energy consumed to the initial mass m (g) of the samples and the ratio of energy consumed to the mass of water W (g) removed from the samples.

The specific energy consumptions of CD, represented as $E_{C_{m}}$ $\left(kJ\;g^{-1}\;fw\right)$ and $E_{C_{W}}$ $\left(kJ\;g^{-1}\;\mathrm{water}\right),$ were determined using Equations (5) and (6), respectively [18].

(5) $$E_{C_{m}}=\frac{E_{C}}{m}$$

(6) $$E_{C_{W}}=\frac{E_{C}}{W}$$

Equations (7) and (8) show the specific energy consumptions of VMD, $E_{VM_{m}}$ $\left(kJ\;g^{-1}\;fw\right)$ and $E_{VM_{W}}$ $\left(kJ\;g^{-1}\;\mathrm{water}\right),$ respectively [18].

(7) $$E_{VM_{m}}=\frac{E_{VM}}{m}$$

(8) $$E_{VM_{m}}=\frac{E_{VM}}{W}$$

The specific energy consumptions of CPD-VMFD, namely, $E_{C-VM_{m}}$ $\left(kJ\;g^{-1}\;fw\right)$ and $E_{C-VM_{W}}$ $\left(kJ\;g^{-1}\;water\right),$ were calculated based on Equations (9) and (10), respectively [18]. Both equations are the ratios of the sum of energy $E_{C}$ and $E_{VM}$ to the mass of the initial sample m (g) and the ratio of the sum of energy $E_{C}$ and $E_{VM}$ to the mass of water (g) from CPD ($W_{C}$) and VMFD ($W_{VM}$) removed from the sample, respectively.

(9) $$E_{C-VM_{m}}=\frac{E_{C}+E_{VM}}{m}$$

(10) $$E_{C-VM_{m}}=\frac{E_{C}+E_{VM}}{W_{C}W_{VM}}\;$$

### 3.7. Colour Analysis

The colour of fresh and dried ground leaf samples was analysed using a Minolta Chroma Meter CR-200 (Minolta Co. Ltd., Osaka, Japan). The data obtained were expressed using L*, a* and b* coordinates. Colour measurements were repeated for five times. In every measurement, the fragmented fresh leaves were placed in different positions, and the ground and dried leaves were mixed in each reading.

### 3.8. Extraction of Polyphenol Compounds

The extraction of polyphenol compounds was carried out based on a method previously described [38]. Approximately 0.3 g of ground samples was measured in test tubes and mixed with 80% aqueous methanol (0.7 mL) and 1% HCl. The suspension was stirred and sonicated twice for 15 min and left to stand at 4 °C for 24 h. The extract was centrifuged at 15,000 rpm (MPW-360R, Warsaw, Poland) for 10 min, and the supernatants were collected thereafter.

### 3.9. Antioxidant Activity Analysis

#### 3.9.1. ABTS^●+^ Radical-Scavenging Assay

The ABTS radical-scavenging assay was performed to determine the free-radical-scavenging activity as described previously [39]. Firstly, ABTS was dissolved in water to obtain a solution of 7 mM concentration. The resultant ABTS solution was mixed with 2.45 mM potassium persulfate to produce ABTS radical cation (ABTS^●+^). This mixture was set aside in the dark at room temperature for 12–16 h prior to its use in the assay. Before the analysis, this solution was diluted with distilled water until an absorbance of 0.700 ± 0.02 at 734 nm is obtained. For the analysis, 300 µL of diluted ABTS radical solution was initially mixed with 20 µL of the extracted supernatant. The absorbance was measured using a UV-vis spectrophotometer (Shimadzu, UV-2401 PC, Kyoto, Japan) at 734 nm after 6 min. Determinations of the absorbance value were performed in triplicate. The results were expressed relative to micromolar Trolox per 100 g of dw in terms of Trolox equivalent antioxidant capacity.

#### 3.9.2. FRAP Assay

FRAP assay was performed as previously described by Benzie and Strain [40]. The FRAP reagent was formed by mixing acetate buffer (300 µM, pH 3.6), 10 µM TPTZ in 40 µM HCl and 20 µM of FeCl_3_ at a ratio of 10:1:1 (*v*/*v*/*v*). Thereafter, 300 µL of the FRAP reagent was mixed with 10 µL of the sample solution. Absorbance was measured using a UV-vis spectrophotometer (Shimadzu, UV-2401 PC, Kyoto, Japan) at 593 nm after 10 min. A standard curve with different concentrations of Trolox was then plotted. Finally, determinations were performed in triplicates.

### 3.10. Total Phenolic Content Analysis

The total phenolic content was determined using the Folin–Ciocalteu method previously described by Gao et al. [41]. A sample extract of 0.1 mL was mixed with 0.2 mL of Folin–Ciocalteu reagent and 2 mL of water. This mixture was incubated at room temperature for 3 min, subsequently added with 1 mL of 20% sodium carbonate and was incubated at room temperature for 1 h. The absorbance was measured using an ultraviolet–visible light spectrophotometer (Shimadzu, UV-2401 PC, Kyoto, Japan) at a wavelength of 765 nm. Quantification was performed on the basis of the gallic acid standard curve constructed, and results were expressed as gallic acid equivalence in milligrams per 100 g of dw. All determinations were performed in triplicate.

### 3.11. Analysis of Volatile Compounds

#### 3.11.1. Headspace Solid-Phase Microextraction (HS-SPME)

Volatiles were extracted using HS-SPME method, reported in a previous study with minor modifications [42]. Approximately 0.25 g of sample was inserted in glass vials with 2 µg of *p*-methoxyphenol as the internal standard and subsequently placed in a laboratory water bath at 60 °C for 10 min for equilibration. SPME fibre, DVB/CAR/PDMS, 50/30 μm, with 2 cm coating (Supelco, Bellefonte, PA, USA) was used for the extraction. The SPME fibre was inserted into the vial with sample that was kept in a water bath at 60 °C for 30 min. Next, the fibre was injected into the injection port of GC–MS at an injection temperature of 220 °C for 3 min for analyte desorption.

#### 3.11.2. GC–MS Analysis of Volatile Compounds

GC–MS analysis was conducted on Varian CP-3800/Saturn 2000 (Varian, Wallnut Creek, CA, USA) using a ZB-5MS capillary column (30 m × 0.25 mm inner diameter (i.d.) × 0.25 μm film thickness). The GC oven temperature was programmed at 50 °C, increased to 130 °C at a rate of 4 °C min^−1^, subsequently raised to 180 °C at 10 °C min^−1^ and finally to 280 °C at a rate of 2 °C min^−1^. Helium was used as a carrier gas at a flow rate of 1.0 mL min^−1^. The samples were injected at a 1:10 split mode. Mass spectra were obtained in an electronic ionisation (EI) mode of 70 eV with a scan range of *m*/*z* 35–550. Identification of all volatile compounds was based on comparing the compound mass spectra obtained experimentally, with the mass spectra available in the NIST14 database. The experimentally determined retention index (RI) by Kovats was also compared with the RI in NIST WebBook and literature data [43]. The quantification analysis of identified volatile compounds was calculated by comparing the peak area of compounds with the peak area of the internal standard (*p*-methoxyphenol) with a concentration of 1 mg/mL.

### 3.12. Phytosterol and Fatty Acid Analysis

#### 3.12.1. Lipid Extraction

The lipid fraction was extracted on the basis of a method described in a previous study with minimal modifications [44]. Ground leaves were extracted using a 2:1 chloroform–methanol (*v*/*v*) for 24 h and filtered thereafter. A vacuum rotary evaporator was used to remove the extraction solvent from the filtrate at 65 °C. The resultant crude lipids were saponified using 10 mL 0.5 M KOH/MeOH at 80 °C for 10 min, boiled with reflux for 10 min and was cooled to 12 °C. The sample was placed in a separation funnel and added with 10 mL of hexane and 10 mL of water, and the mixture was mixed vigorously. Fatty acid fraction that was partitioned to the polar phase was segregated from the phytosterol fraction.

#### 3.12.2. GC–MS Analysis of Phytosterols

Phytosterol portion that was obtained in Section 3.9.1 was dried with MgSO_4_, and the extraction solvent was evaporated using a vacuum rotary evaporator. The resultant residue was added with 0.2 mL of pyradine and 0.2 mL of BSTFA as a silylation agent. Approximately 1 mg of cholesterol was added as the internal standard. This mixture was placed in an incubator orbital shaker at 110 rpm and heated at 60 °C for 45 min. Thereafter, this mixture was transferred to small tubes for GC–MS analysis. Identification of phytosterols was based on comparing the experimentally obtained mass spectra with the mass spectra available in literature and by comparing the relative retention times of the standards. The concentrations of identified phytosterols were obtained by comparing the peak area of each compound to that of the internal standard cholesterol with a concentration of 1 mg/mL.

#### 3.12.3. GC–MS Analysis of Fatty Acids

The fatty acid portion obtained in Section 3.9.1 was acidified with 1 M HCl and was extracted with 10 mL of hexane. The organic portion was segregated, whereas the extraction solvent was removed with a vacuum rotary evaporator. Methylation was performed by adding 4 mL of 14% BF_3_/MeOH (*v*/*v*) at 80 °C for 10 min. The produced fatty acid methyl ester (FAME) was subsequently extracted with 2.5 mL of hexane, dried using MgSO_4_ and finally filtered with a cotton plug and silicate powder. The FAME profile was analysed using a GC–MS equipment (GCMS-QP 2020, Shimadzu, Kyoto, Japan). Separation was performed using a Zebron ZB-WAX capillary column (30 m × 0.25 mm i.d. × 0.25 μm film thickness; Phenomenex, Torrance, CA, USA). The scanning was conducted at a mass range of 50–400 *m*/*z* at an EI of 70 eV and at a 5 scan s^−1^ mode. The carrier gas used was helium with a flow rate of 1.0 mL min^-–1^ at a split ratio of 1:10. The temperature gradient programmes used were as follows: (a) 45 °C for 3 min, (b) 45 °C to 220 °C at a rate of 5 °C min^−1^ and (c) 220 °C–250 °C at a rate of 10 °C min^−1^ and finally maintained at 250 °C for 2 min. The injector was held at a temperature of 260 °C.

### 3.13. Statistical Analysis

One-way analysis of variance (ANOVA) was performed using SPSS version 20 (IBM, Tulsa, OK, USA). The results were presented as means ± standard deviation. Significant differences (*p* ≤ 0.05) between the mean values were evaluated with Tukey’s test. Mathematical modelling was performed using Table Curve 2D Windows v2.03 (Jandel Scientific Software, USA). The RMSE and coefficient of determination *R^2^* were determined to know the goodness of fit of the mathematical models.

## 4. Conclusions

The drying kinetics of *C. alata* leaves dehydrated using CD, VMD and CPD-VMFD was best described by the exponential model (modified Page model). The antioxidant activity, TPC and total volatile concentration did not improve with the use of advanced drying methods (VMD and CPD-VMFD), as CD at 40 °C achieved the highest antioxidant activity, TPC and total volatile concentration. However, CPD-VMFD is effective in ensuring a high phytosterol concentration than CD at 50 °C. Drying of *C. alata* leaves with VMD required the least energy than conventional CD and CPD-VMFD. However, CPD-VMFD is more efficient in energy saving because this combined drying method reduced the final energy consumption by 32.82% (kJ/g fresh weight) and 26.12% (kJ/g water) compared with CD at 50 °C. FD produced dried *C. alata* with best the colour properties whilst CD at 60 °C produced sample with lowest a_w_. Based on the results of this study, the final recommendation of a suitable drying method to dehydrate *C. alata* leaves is CD at 40 °C, considering the antioxidant activity, TPC and total volatiles yield.

## Figures and Tables

**Table 1:** Final moisture content and water activity of dried *C. alata* dehydrated using different methods.

Drying Method	Final Moisture Content (kg water/ kg dw)	Water Activity *a_w_*
FD	0.0575 ± 0.001	0.2836 ± 0.000 b
CD (40 °C)	0.0372 ± 0.000	0.2258 ± 0.010 c
CD (50 °C)	0.0336 ± 0.001	0.1558 ± 0.004 f
CD (60 °C)	0.0289 ± 0.001	0.1291 ± 0.004 g
VMD (6 W/g)	0.0372 ± 0.003	0.1905 ± 0.002 d
VMD (9 W/g)	0.0347 ± 0.002	0.1697 ± 0.005 e,f
VMD (12 W/g)	0.0423 ± 0.002	0.2200 ± 0.003 c
CPD-VMFD	0.0343 ± 0.002	0.1726 ± 0.004 e

FD—freeze drying; CD—convective drying, VMD—vacuum microwave drying, CPD—convective pre-drying, VMFD—vacuum microwave finish drying. Values with the same letter in the same column are not significantly different (*p* < 0.05), according to Tukey’s test.

**Table 2:** Colour parameters L*, a* and b* of *C. alata* influenced by different drying methods.

Drying Conditions	Colour Parameters		
	L*	a*	b*
Fresh	36.94 ± 0.093 a	−2.75 ± 0.451 a	9.04 ± 0.440 a
FD	44.71 ± 0.322 b	−2.17 ± 0.064 b	17.00 ± 0.320 b
CD (40 °C)	38.55 ± 0.095 b	−0.20 ± 0.118 d,e	10.65 ± 0.109 c
CD (50 °C)	38.75 ± 0.070 b	0.00 ± 0.056 e,f	10.53 ± 0.101 c
CD (60 °C)	38.31 ± 0.207 e	0.39 ± 0.025 g	10.04 ± 0.186 d
VMD (6 W/g)	40.27 ± 0.036 d	0.45 ± 0.044 g	13.81 ± 0.125 e
VMD (9 W/g)	39.42 ± 0.388 c	−0.47 ± 0.051 c,d	11.89 ± 0.143 f
VMD (12 W/g)	39.43 ± 0.272 c	−0.54 ± 0.042 c	12.25 ± 0.115 f
CPD-VMFD	39.30 ± 0.110 c	0.18 ± 0.055 f,g	11.82 ± 0.149 f

FD—freeze drying; CD—convective drying; VMD—vacuum microwave drying; CPD—convective pre-drying; VMFD—vacuum microwave finish drying; values with the same letter within a column are not significantly different (*p* < 0.05), according to Tukey’s test.

**Table 3:** Antioxidant activity and TPC of *C. alata,* influenced by various drying methods and conditions.

Drying Method	Antioxidant Activity (µM Trolox/100 g dw)		Total Phenolic Content (mg/100 g dw)
	ABTS	FRAP	
Fresh	65.53 ± 4.97 a	30.69 ± 6.91 a	9368.59 ± 1460.98 a
FD	11.49 ± 0.25 b,c	5.34 ± 0.17 b	2883.05 ± 146.45 b
VMD 6 W/g	9.04 ± 1.19 b,c	3.28 ± 0.08 b	1982.44 ± 53.17 b
VMD 9 W/g	11.29 ± 1.03 b,c	4.70 ± 0.25 b	2379.87 ± 216.92 b
VMD 12 W/g	9.15 ± 1.70 b,c	5.39 ± 0.38 b	2441.91 ± 82.86 b
CPD-VMFD	11.19 ± 0.33 b,c	5.01 ± 0.18 b	2413.36 ± 277.94 b
CD at 40 °C	13.54 ± 0.02 c	5.55 ± 0.24 b	2965.12 ± 54.97 b
CD at 50 °C	8.65 ± 0.27 b,c	3.88 ± 0.58 b	2342.75 ± 74.18 b
CD at 60 °C	7.43 ± 0.63 b	2.58 ± 0.09 b	1821.54 ± 41.18 b

FD—freeze drying; CD—convective drying, VMD—vacuum microwave drying, CPD—convective pre-drying, VMFD—vacuum microwave finish drying. Values with the same letter in the same column are not significantly different (*p* < 0.05) according to Tukey’s test.

**Table 4:** Concentration of volatile compounds influenced by various drying methods and conditions.

Compound	RT	Retention Indexes		Concentration (mg 100 g^−1^ db)								
		Exp	Lit	Fresh	FD	CD (40 °C)	CD (50 °C)	CD (60 °C)	VMD (6 W/g)	VMD (9 W/g)	VMD (12 W/g)	CPD-VMFD
2-Hexenal	4.770	843	851	60.28	2.98	7. 19	5.57	6.71	0.45	1.07	4.89	2.13
2-Hexen-1-ol, (E)-	5.030	858	862	10.11	0.65	0.82	0.50	0.38	0.00	0.18	1.66	0.31
1-Hexanol	5.070	861	868	18.70	1.85	0.78	0.62	0.80	0.02	0.95	6.37	0.43
2-Heptanol	5.810	899	901	1.62	0.33	1.37	0.58	0.80	0.16	0.20	1.11	0.37
Anisole	6.330	917	920	1.29	0.47	0.38	0.17	0.11	0.01	0.03	0.24	0.09
Benzaldehyde	7.500	959	962	8.37	0.91	1.99	0.71	0.65	0.10	0.21	1.10	0.31
1-Octen-3-ol	8.180	883	880	2.75	0.24	1.67	1.02	0.07	0.00	0.18	1.15	0.04
Phenol	8.100	980	980	5.02	1.02	0.96	0.32	0.09	0.08	0.38	1.00	0.24
5-Hepten-2-one, 6-methyl-	8.260	986	986	4.62	1.08	3.46	2.40	2.12	0.48	1.34	7.50	1.18
3-Octanol	8.430	992	994	3.68	0.46	1.01	0.76	1.10	0.19	0.23	1.60	0.34
5-Hepten-2-ol, 6-methyl-	8.570	997	994	1.19	0.01	0.07	0.02	0.07	0.00	0.10	0.91	0.00
Hexanoic acid, ethyl ester	8.670	1000	1000	2.21	1.33	2.47	0.51	1.27	0.16	0.42	2.63	0.16
Octanal	8.750	1002	1003	0.50	0.53	0.23	0.27	0.41	0.12	0.06	0.24	0.02
Anisole, o-methyl-	9.000	1010	1009	0.95	0.05	0.28	0.12	0.16	0.02	0.06	0.31	0.05
Acetic acid, hexyl ester	9.100	1013	1011	0.36	0.06	0.02	0.00	0.00	0.01	0.01	0.07	0.00
4-Hepten-1-ol, 6-methyl-	9.230	1017	1020	3.36	0.14	0.35	0.36	0.29	0.01	0.04	0.16	0.18
3-Ethyl-4-methylpentan-1-ol	9.320	1020	1023	8.47	0.50	2.11	0.78	0.87	0.06	0.15	0.59	0.35
p-Cymene	9.470	1024	1025	1.18	0.04	0.68	0.14	0.17	0.09	0.10	0.37	0.07
Limonene	9.610	1029	1030	2.18	0.05	0.22	0.15	0.13	0.05	0.17	1.25	0.41
Eucalyptol	9.700	1031	1032	1.92	0.00	0.00	0.02	0.01	0.00	0.00	0.02	1.88
Benzyl alcohol	9.800	1035	1036	13.42	2.92	4.67	2.81	2.86	0.49	1.22	4.36	0.01
Benzeneacetaldehyde	10.070	1042	1045	3.28	0.96	0.89	0.48	0.63	0.06	0.37	0.98	0.28
Ether, benzyl ethyl	10.500	1056	1046	2.51	0.24	0.17	0.08	0.12	0.02	0.13	0.14	0.02
Fenchone	11.580	1088	1096	7.99	0.22	0.57	0.16	0.02	0.02	0.04	1.18	0.05
2-Nonanone	11.660	1091	1092	1.01	0.08	0.40	0.02	0.10	0.00	0.04	0.32	0.04
Ethyl (4E)-4-heptenoate	11.740	1094	1090	3.63	0.64	1.03	0.53	0.67	0.04	0.06	0.26	0.01
Linalool	11.960	1100	1099	10.59	0.75	2.95	1.26	1.20	0.13	0.33	1.80	0.46
Nonanal	12.080	1104	1104	2.81	0.16	0.91	0.45	0.86	0.09	0.27	0.97	0.15
β-Thujone	12.180	1107	1114	1.12	0.70	0.66	0.44	0.59	0.07	0.39	1.11	0.20
Phenylethyl alcohol	12.440	1114	1116	1.82	0.42	3.90	0.80	0.83	0.00	0.20	1.60	0.08
3-Thujanone	12.560	1118	1119	1.01	0.69	0.66	1.01	1.17	0.09	0.67	2.20	0.15
Veratrol	13.540	1147	1148	6.49	0.47	1.65	0.44	0.54	0.10	0.14	0.62	0.23
2,6-Nonadienal, (E,Z)-	13.770	1153	1155	5.75	0.21	0.72	0.24	0.32	0.01	0.08	0.13	0.12
2-Nonenal, (E)-	14.000	1160	1162	0.39	0.34	1.48	0.68	0.71	0.09	0.20	0.00	0.36
endo-Borneol	14.260	1167	1167	0.32	0.31	1.13	0.39	0.66	0.10	0.06	0.25	0.29
Hexanoic acid, butyl ester	15.070	1191	1189	1.20	0.03	0.06	0.04	0.08	0.01	0.02	0.15	0.03
Methyl salicylate	15.200	1195	1192	5.18	0.27	1.18	0.06	0.78	0.17	0.02	0.23	0.38
2-Octynoic acid, methyl ester	15.550	1210	1212	2.91	0.16	0.73	0.49	0.50	0.04	0.15	0.53	0.25
Citronellol	16.330	1228	1228	13.26	0.93	2.96	1.28	1.91	0.02	0.20	0.06	0.81
Butanoic acid, 2-methyl-, hexyl ester	16.650	1238	1236	1.56	0.21	1.19	0.38	0.49	0.07	0.21	0.70	0.23
D-Carvone	16.900	1245	1246	0.65	0.22	0.90	0.27	0.32	0.06	0.13	0.80	0.19
Salicylic acid, ethyl ester	17.850	1274	1270	15.05	0.58	1.32	0.61	0.60	0.07	0.15	0.70	0.27
2-Undecanone	18.480	1293	1294	2.62	0.01	2.13	0.17	0.48	0.06	0.03	0.51	0.01
Nonanoic acid, ethyl ester	18.620	1296	1296	0.55	0.01	0.15	0.01	0.05	0.00	0.00	0.04	0.00
Methyl 4-methylsalicylate	19.270	1319	-	1.86	0.17	0.28	0.15	0.09	0.02	0.15	0.18	0.08
β-Cubebene	21.400	1391	1389	2.54	0.08	1.38	0.56	0.87	0.04	0.28	0.84	0.35
Geranyl acetone	22.800	1457	1453	0.86	0.10	0.33	0.18	0.25	0.04	0.12	0.35	0.13
trans-β-Ionone	23.440	1489	1486	2.59	0.24	0.80	0.48	0.51	0.07	0.27	0.78	0.23
Widdrol	25.450	1618	1610	1.92	0.02	0.08	0.03	0.00	0.00	0.01	0.04	0.01
Isopropyl myristate	27.400	1825	1827	1.10	0.05	0.53	0.22	0.13	0.07	0.11	0.68	0.06
TOTAL				254.79 a	24.91 c	61.85 b	29.73 c	34.55 c	11.91 d	11.93 d	55.67 b	14.04 c,d

RT—retention time; RI—retention index; Exp—experimental; Lit—literature; FD—freeze drying; CD—convective drying; VMD—vacuum microwave drying; CPD—convective pre-drying; VMFD—vacuum microwave finish drying; nd—not detected. ^a^ NS, not significant F ratio (*p* < 0.05); ^b^ Treatment means of the ANOVA test; Values followed by the same letter, within the same row, are not significantly different (*p* < 0.05), according to Tukey’s multiple-range test.

**Table 5:** Concentration of phytosterols influenced by drying methods.

Compound	Retention time	ANOVA	Fresh	CPD-VMFD	CD at 50 °C
			Concentration (mg 100 g^−1^ db)		
α-tocopherol	25.945	n.s.^a^	106.08 a	106.32 a	85.85 a,b
Desmosterol	26.630	***	218.08 a	37.74 b	42.23 b
Lanosterol	26.875	***	152.20 a	19.84 b	22.33 b
Campesterol	27.575	***	498.76 a	128.45 b	132.60 b
Stigmasterol	28.035	***	1001.48 a	221.47 b	193.81 b
β-sitosterol	28.950	***	1162.24 a	282.31 b	264.62 b
β-amyrin	29.195	***	268.82 a	2.78 b	2.31 b
Cycloartenol	30.045	***	67.20 a	0.00 b	0.93 b
Betulin	31.250	***	176.62 a	10.64 b	12.38 b
TOTAL			3647.48 a	809.56 b	757.07 b

CPD—convective pre-drying; VMFD—vacuum microwave finish drying; CD—convective drying.^a^ NS, not significant F ratio (*p* < 0.05); ^b^ Treatment means of the ANOVA test (values are the mean value of three replicates); values followed by the same letter, within the same row, is not significantly different (*p* < 0.05) according to Tukey’s multiple-range test.

**Table 6:** Fatty acid composition of *C. alata*.

Compound	Retention Time	Total Area %
Lauric acid	23.505	0.16 ± 0.11
Myristic acid	27.805	1.34 ± 0.31
Pentadecanoic acid	29.820	0.24 ± 0.08
Palmitic acid	31.745	20.59 ± 5.7
Palmitoleic acid	32.135	2.03 ± 0.37
Hexadecenoic acid, methyl ester, (11Z)-	32.655	0.91 ± 0.15
Heptadecanoic acid	33.590	0.49 ± 0.09
cis-10-Heptadecenoic acid	33.925	0.18 ± 0.05
Stearic acid	35.365	4.47 ± 0.8
Oleic acid	35.640	8.28 ± 0.21
Elaidic acid	35.760	0.40 ± 0.14
Linoleic acid	36.375	23.07 ± 3.12
α-Linolenic acid	37.410	34.78 ± 5.17
Arachidic acid	38.675	0.97 ± 0.31
Behenic acid	41.160	1.86 ± 0.65
cis-4,7,10,13,16,19- Docosahexaenoic acid	42.345	0.23 ± 0.19

**Table 7:** Mathematical models applied to drying curves of *C. alata*.

Model Name	Model Equation
Lewis	*MR* = exp(−*k*·t)
Modified Page	$MR=a\cdot \exp\left(-k\cdot t^{n}\right)$
Henderson and Pabis	MR=a·exp(−k·t)

*MR*—moisture ratio*; a*—coefficient of the equation; *k*—drying constant (min^−1^); *n*—exponent; *t*—time (min).
